# HPIP is upregulated in colorectal cancer and regulates colorectal cancer cell proliferation, apoptosis and invasion

**DOI:** 10.1038/srep09429

**Published:** 2015-03-24

**Authors:** Yingying Feng, Xiaojie Xu, Yunjing Zhang, Jianhua Ding, Yonggang Wang, Xiaopeng Zhang, Zhe Wu, Lei Kang, Yingchun Liang, LiYing Zhou, Santai Song, Ke Zhao, Qinong Ye

**Affiliations:** 1 Affiliated Hospital of Academy of Military Medical Sciences, Beijing, People's Republic of China; 2 Department of Medical Molecular Biology, Beijing Institute of Biotechnology, Beijing, People's Republic of China; 3 Department of Colorectal Surgery, the Second Artillery General Hospital, Beijing, People's Republic of China; 4 Department of Traditional Chinese Medicine, the Second Artillery General Hospital, Beijing, People's Republic of China; 5 Laboratory of Vaccine and Antibody Engineering, Beijing Institute of Biotechnology, Beijing, People's Republic of China; 6 Department of Nuclear Medicine, Peking University First Hospital, Beijing 100034, China

## Abstract

Hematopoietic pre-B cell leukemia transcription factor (PBX)-interacting protein (HPIP) was shown to play a role in cancer development and progression. However, the role of HPIP in colorectal cancer (CRC) is unknown. Here, we report that HPIP is overexpressed in most of CRC patients and predicts poor clinical outcome in CRC. HPIP promotes CRC cell proliferation via activation of G1/S and G2/M checkpoint transitions, concomitant with a marked increase of the positive cell cycle regulators, including cyclin D1, cyclin A, and cyclin B1. HPIP inhibits CRC cell apoptosis accompanied by the decreased levels of BAX and PIG3, the inducers of apoptosis, and the increased level of the apoptosis inhibitor BCL2. HPIP blocks caspase-3-mediated cleavage of PARP, an important apoptosis marker. HPIP promotes CRC cell migration and invasion, and regulates epithelial-mesenchymal transition (EMT), which plays a critical role in cancer cell migration and invasion. Activation of MAPK/ERK1/2 and PI3k/AKT pathways is required for HPIP modulation of CRC cell proliferation, migration and EMT. Moreover, HPIP knockdown suppresses colorectal tumor growth in nude mice. These data highlight the important role of HPIP in CRC cell proliferation and progression and suggest that HPIP may be a useful target for CRC therapy.

Colorectal cancer (CRC) is one of the most common malignancies in the world and the third most frequent cause of cancer-related death in western societies, accounting for approximately 10% of all cancer incidence and mortality^1^. Thus, elucidation of the molecular mechanisms underlying CRC tumorigenesis and progression is critical to individual treatment of CRC. Although it is widely accepted that CRC is a heterogeneous disease defined by different activating mutations in receptor tyrosine kinases (RTKs) or other mutations in downstream components of RTK-activated intracellular pathways^2^, our understanding of the genetic alteration underlying the development of colorectal cancer remains limited.

Hematopoietic pre-B-cell leukemia transcription factor (PBX)-interacting protein (HPIP/PBXIP1), a co-repressor for the transcription factor PBX, is involved in organogenesis and tumorigenesis^3^. We and others have previously shown that HPIP can promote breast cancer cell proliferation via interaction with estrogen receptor (ER)^4^,^5^. HPIP is overexpressed in breast infiltrative ductal carcinoma^6^ and astrocytoma^7^ and promotes proliferation and migration of astrocytoma cells^7^. Our recent studies reveal that HPIP is overexpressed in most of 328 liver cancer patients and regulates hepatoma cell proliferation^8^. However, the association between HPIP and colorectal cancer (CRC) remains unclear.

In this study, we show that expression of HPIP is higher in CRC tissues than matched non-cancerous tissues and predicts bad clinical outcome. HPIP promotes CRC cell proliferation, migration and invasion, while it inhibits apoptosis of CRC cells. HPIP knockdown reduces colorectal tumor growth in nude mice. Moreover, HPIP regulates these events through changes in expression of corresponding genes.

## Results

### Upregulation of HPIP expression in CRC patients

**Figure 1:**
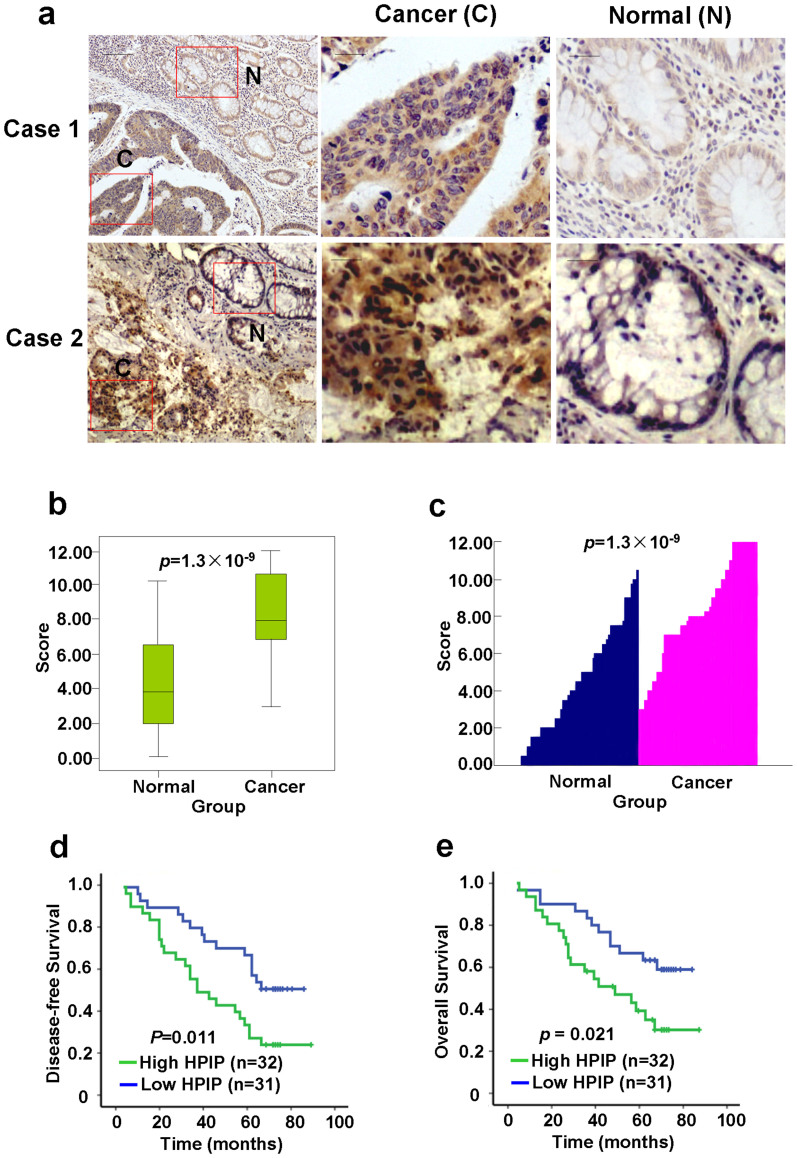
HPIP expression is upregulated in CRC patients. (A) Representative immunohistochemical staining of HPIP protein in colorectal cancer tissue (middle) and matched adjacent normal colorectal tissue (right). The boxed areas in the left images are magnified in the middle and right images. Scale bar: 200 μm (left), 50 μm (middle and right). (B and C) HPIP expression scores were displayed in box-and-whisker plots (B) and bar chart (C), and compared using Mann-Whitney *U* test. (D and E) Kaplan-Meier estimates of disease-free survival (D) and overall survival (E) of CRC patients. Marks on graph lines represent censored samples.

We detected the expression of HPIP by IHC on tissues consisting of 63 pairs of human colorectal tumors and matched non-tumor colorectal tissues. HPIP was distributed mainly in the cytoplasm. Based on HPIP scores, HPIP expression was significantly upregulated in CRC patients (*p* = 1.3 × 10^−9^) (Fig. 1A–C). Focusing on paired tumor and normal tissues, in 92.1% (58/63) of patients, the expression levels of HPIP in tumors were higher than those in adjacent normal tissues; in 7.9% (5/63) of patients, the cancers expressed lower levels of HPIP than normal tissues. Moreover, the patients with high expression of HPIP had shorter disease-free survival (DFS) and overall survival (OS) than those with low expression of HPIP (DFS: *p* = 0.011, OS: *p* = 0.021) (Fig. 1D, 1E), indicating that HPIP predicts poorer clinical outcome of CRC. The specificity of anti-HPIP antibody was confirmed by immunohistochemical staining of CRC tissues incubated with anti-HPIP preincubated with its antigen (Supplementary Fig. 1A) and immunoblotting of lysates from HCT-8 and SW480 CRC cells infected with HPIP shRNA (Supplementary Fig. 1B).

### HPIP promotes colorectal cancer cell proliferation in vitro

**Figure 2:**
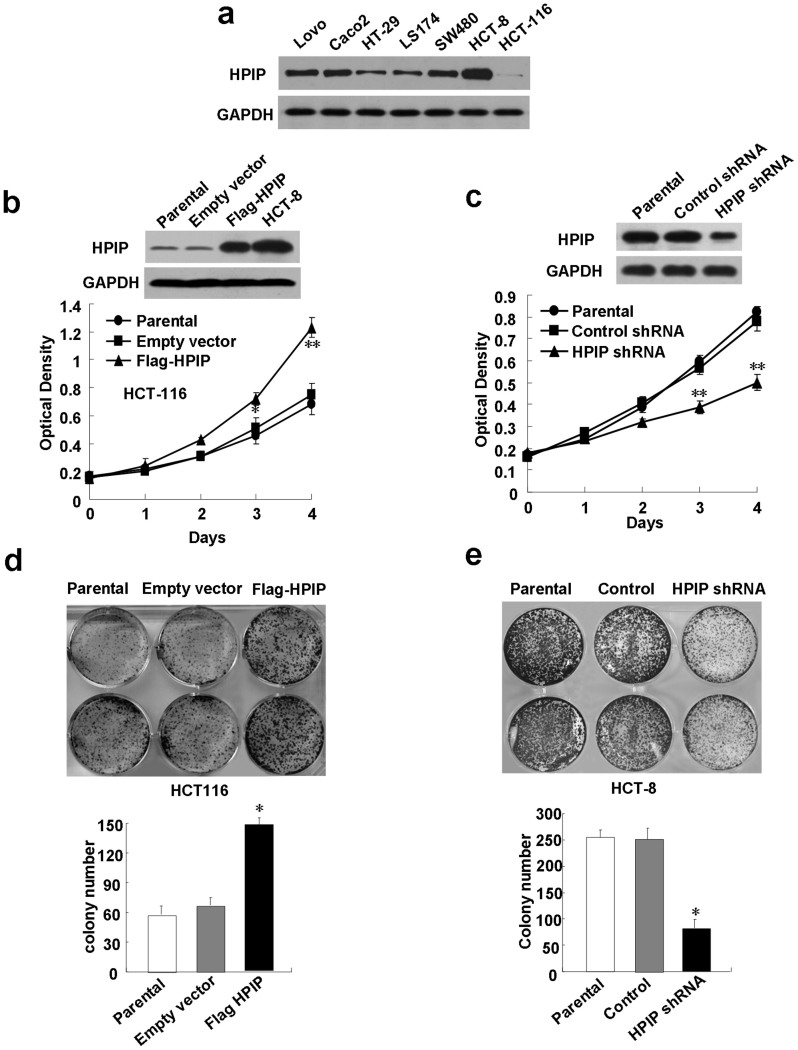
HPIP promotes CRC cell proliferation. (A) Total proteins extracted from the indicated CRC cell lines were analyzed by immunoblotting with anti-HPIP. GAPDH was used as a loading control. (B) HCT-116 cells transfected with Flag-tagged HPIP or empty vector or parental HCT-116 cells were grown in regular medium and harvested at the indicated times. Cell number was determined by CCK-8 assay. The representative immunoblot with anti-HPIP indicates HPIP expression levels. HCT-8 cells were used for comparison of HPIP expression in different groups. (C) HCT-8 cells infected with HPIP shRNA were cultured and analyzed as in (B). (D) Colony formation assays for HCT-116 cells transfected as in (B). (E) Colony formation assays for HCT-8 cells infected as in (C). All values shown are mean ± SD of triplicate measurements and have been repeated 3 times with similar results (*p < 0.05 versus empty vector or control shRNA, **p < 0.01 versus empty vector or control shRNA).

Next, the effect of HPIP overexpression or knockdown of endogenous HPIP protein on anchorage- dependent growth of CRC cells was investigated. All seven CRC cell lines (Lovo, Caco2, HT-29, LS174, SW480, HCT-8, and HCT-116) expressed endogenous HPIP protein (Fig. 2A & Supplementary Fig. 5A, 5B). Among them, HCT-8 cell line expressed HPIP at the highest level, HCT-116 cell line expressed HPIP at the lowest level, and SW480 cell line expressed HPIP at the medium level. Therefore, to study the role of HPIP in CRC, we chose HCT-8 to knockdown HPIP, HCT-116 to overexpress HPIP, and SW480 to overexpress and knockdown HPIP. HCT-116 cells transfected with FLAG-tagged HPIP grew much faster than those transfected with empty vector or parental HCT-116 cells (Fig. 2B & Supplementary Fig. 5C, 5D). Although HPIP was overexpressed in HCT-116 cells, the expression level of HPIP was still lower than that of endogenous HPIP in HCT-8 cells, suggesting physiological role of HPIP in regulation of CRC cell growth. Similar results were obtained in SW480 cells infected with HPIP-expressing lentivirus (Supplementary Fig. 2A). In contrast, HCT-8 and SW480 cells infected with HPIP shRNA grew more slowly than those infected with control shRNA or their parental cells, indicating that HPIP knockdown in HCT-8 and SW480 cells reduces cell proliferation (Fig. 2C & Supplementary Fig. 2B & Supplementary Fig. 5E, 5F). Moreover, colony formation assays revealed that colony number and colony size were larger in HPIP-overexpressing HCT-116 and SW480 cells than in empty vector-containing cells or their parental cells (Fig. 2D & Supplementary Fig. 2C), while knockdown of HPIP with HPIP shRNA in SW480 and HCT-8 cells decreased the colony number and size (Fig. 2E & Supplementary Fig. 2D). These results reveal that HPIP increases the proliferation and colony formation of CRC cells.

### HPIP activates the G1/S and G2/M transitions in CRC cells

**Figure 3:**
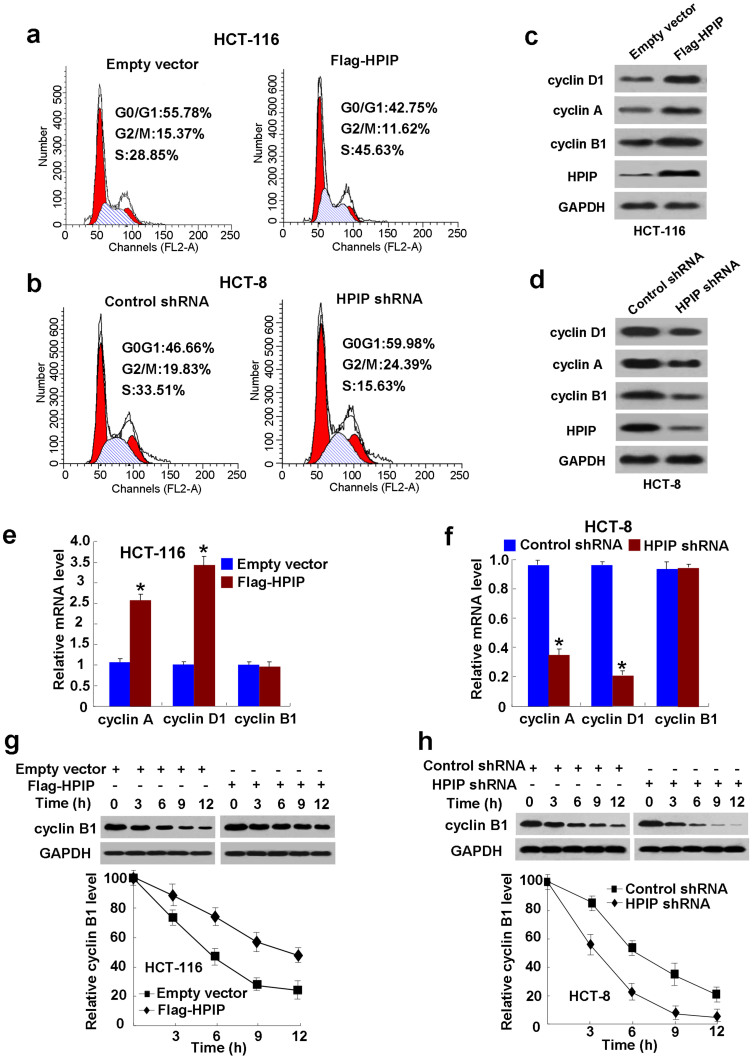
HPIP activates the G1/S and G2/M transitions in CRC cells. (A) Flow cytometry analysis of cell cycle in HCT-116 cells transfected with empty vector or Flag-HPIP. (B) Flow cytometry analysis of cell cycle in HCT-8 cells infected with control shRNA or HPIP shRNA. The experiments have been repeated three times with similar trends and the image displayed is one of the representative results. (C and D) Representative Western blots for cyclin D1, cyclin A and cyclin B1 proteins in HPIP-overexpressing HCT-116 cells (C) and HPIP knockdown HCT-8 cells (D). (E and F) RT-qPCR analyses for cyclin D1, cyclin A and cyclin B1 mRNA expression in HPIP-overexpressing HCT-116 cells (E) and HPIP knockdown HCT-8 cells (F). Data shown are mean ± SD of 3 independent experiments. *p < 0.05 versus corresponding empty vector or control shRNA. (G and H) Immunoblot analysis of cyclin B1 expression in HPIP-overexpressing HCT-116 cells (G) and HPIP knockdown HCT-8 cells (H) at the indicated times after exposure to the protein synthesis inhibitor cycloheximide (20 μg/ml). Graphs show quantification of immunoblot data. Data shown are mean ± SD of 3 independent experiments.

To elucidate the mechanism how HPIP promotes CRC cell growth, we investigated the effect of HPIP on cell cycle distribution by flow cytometry analysis. Compared with the control cells, overexpression of HPIP in HCT-116 cells resulted in a reduction in the proportion of cells in G0/G1 phase (from 55.78% to 42.75%) and G2/M phase (from 15.37% to 11.62%) but an increase in the proportion of cells in S-phase (from 28.85% to 45.63%) (Fig. 3A). In contrast, knockdown of HPIP in HCT-8 cells significantly increased the proportion of cells in both G0/G1 (46.66% to 59.98%) and G2/M (19.83% to 24.39%) phases, which associated with decreased proportion of cells in S-phase (33.51% to 15.63%) (Fig. 3B). These data suggest that HPIP activates both the G1/S and the G2/M transitions in CRC cells.

### HPIP regulates the expression of G1 and G2 phase-related proteins in CRC cells

Since HPIP regulates cell cycle distribution, we examined the expression of several important cell cycle-related proteins in HPIP knockdown or overexpressing CRC cells. Overexpression of HPIP in HCT-116 cells increased the expression of the G1/S-phase markers cyclin D1 and cyclin A as well as the G2/M-phase marker cyclin B1 (Fig. 3C & Supplementary Fig. 5G–K). On the contrary, knockdown of endogenous HPIP in HCT-8 cells decreased the expression of cyclin D1, cyclin A and cyclin B1 (Fig. 3D & Supplementary Fig. 5L–P).

To investigate the mechanism of how HPIP regulates cyclins, we performed RT-qPCR to test the effects of HPIP on the mRNA levels of cyclin D1, cyclin A, and cyclin B1. HPIP overexpression increased cyclin A and cyclin D1 mRNA levels in HCT-116 cells, while knockdown of HPIP decreased cyclin A and cyclin D1 mRNA levels in HCT-8 cells (Fig. 3E, 3F). Moreover, HPIP did not alter the mRNA level of cyclin B1 in these CRC cells (Fig. 3E, 3F). These data suggest that HPIP regulates cyclin A and cyclin D1 expression at the transcriptional level, while it regulates cyclin B1 expression at the posttranscriptional level.

Next, we used promoter luciferase reporter assay to further confirm if HPIP regulates cyclin A and cyclin D1 transcription. We found that HPIP overexpression significantly increased the promoter activities of cyclin A and cyclin D1 (Supplementary Fig. 3A, 3B).

Since HPIP modulates cyclin B1 expression at the posttranscriptional level, we determined the effect of HPIP on the half-life of cyclin B1 protein. Cycloheximide (CHX) chase assay showed that HPIP overexpression increased the half-life of cyclin B1 protein from approximately 6 h to 12 h (Fig. 3G & Supplementary Fig. 5Q–T), whereas HPIP knockdown decreased that of cyclin B1 protein from approximately 6 h to 3.5 h (Fig. 3H & Supplementary Fig. 5U–X). The finding that HPIP regulates cyclin B1 protein stability suggests that the ubiquitin-proteasome pathway may be involved in this process. Indeed, addition of the proteasome inhibitor MG132 blocked HPIP knockdown-mediated cyclin B1 degradation (Supplementary Fig. 3C), suggesting that the ubiquitin-proteasome pathway is involved in HPIP modulation of cyclin B1 protein stability.

### HPIP inhibits CRC cell apoptosis

**Figure 4:**
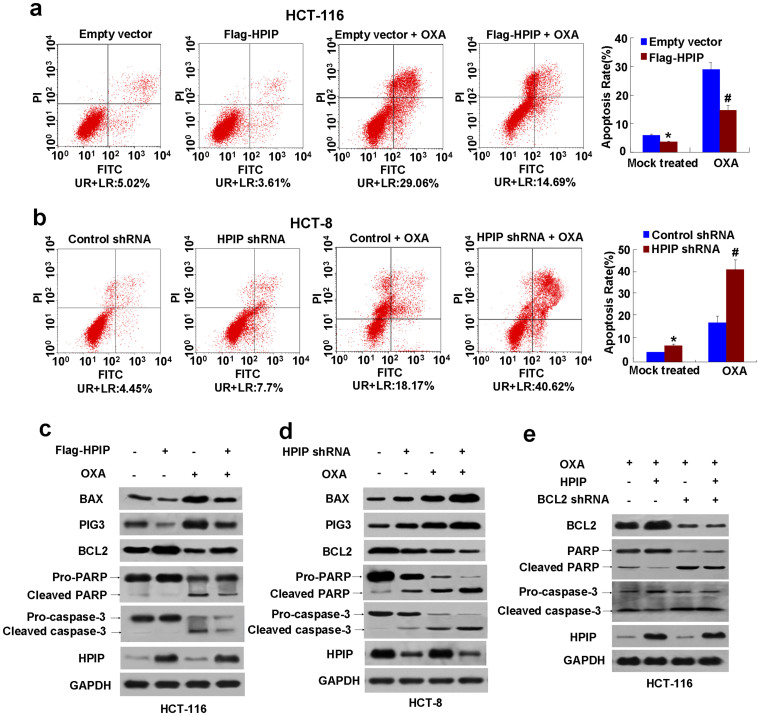
HPIP inhibits CRC cell apoptosis. (A and B) Representative flow cytometry analysis of apoptosis stained with Annexin V and PI in (A) HCT-116 cells transfected with Flag-HPIP or (B) HCT-8 cells infected with HPIP shRNA, treated with or without Oxaplatin (OXA). Data shown are mean ± SD of three independent experiments (*p < 0.05 versus empty vector or control shRNA without OXA, #*p* < 0.05 versus empty vector or control shRNA with OXA). (C and D) Representative Western-blots with the indicated antibodies in (C) HCT116 and (D) HCT-8 cells transfected/infected and treated as in (A) and (B). (E) Representative Western-blots with the indicated antibodies in BCL2 knockdown HCT-116 cells or control cells transfected with Flag-HPIP or empty vector in the presence of OXA.

Next, we determined whether HPIP can regulate apoptosis of CRC cells. The percentage of apoptotic cells was lower in the HPIP-overexpressing HCT-116 cells than that in the empty vector-containing HCT-116 cells (from 5.02% to 3.61%) (Fig. 4A). In contrast, HPIP knockdown in HCT-8 cells showed higher percentage of apoptosis (from 4.45% to 7.7%) (Fig. 4B). Interestingly, based on apoptosis, HPIP overexpression reduced sensitivity of HCT-116 cells to Oxaplatin, a chemotherapy drug used for treatment of CRC, whereas HPIP knockdown increased sensitivity of HCT-8 cells to Oxaplatin, because the altered amplitude of HPIP-regulated apoptosis with Oxaplatin was different from that without Oxaplatin (Fig. 4A, 4B). Consistent with the results of HPIP modulation of apoptosis, HPIP overexpression decreased the expression of BAX and PIG3, the inducers of apoptosis, and increased the expression of the apoptosis inhibitor BCL2 (Fig. 4C & Supplementary Fig. 6A–C). Opposite results were observed in HPIP knockdown cells (Fig. 4D & Supplementary Fig. 6H–J). Downstream of the BCL2 and BAX proteins in the apoptotic cascade are the caspases. caspase-3 is a critical executioner of apoptosis and poly (ADP-ribose) polymerase (PARP) is the caspase-3 substrate. Interestingly, HPIP overexpression revealed decreased cleaved (active) caspase-3 and cleaved PARP (Fig. 4C & Supplementary Fig. 6D, 6E), whereas HPIP knockdown showed increased cleaved caspase-3 and cleaved PARP (Fig. 4D & Supplementary Fig. 6K, 6L).

To investigate how HPIP inhibits Oxaplatin-mediated apoptosis, we determined if HPIP regulates apoptosis via regulation of apoptosis-related proteins by silencing BCL2, one of the key regulators of apoptosis. The results demonstrated that the inhibitory effect of HPIP on Oxaplatin-mediated apoptosis was greatly impaired when BCL2 was knocked down (Fig. 4E & Supplementary Fig. 6O–R), suggesting that BCL2 is critical for HPIP modulation of apoptosis.

### HPIP promotes CRC cell migration and invasion with increased EMT

**Figure 5:**
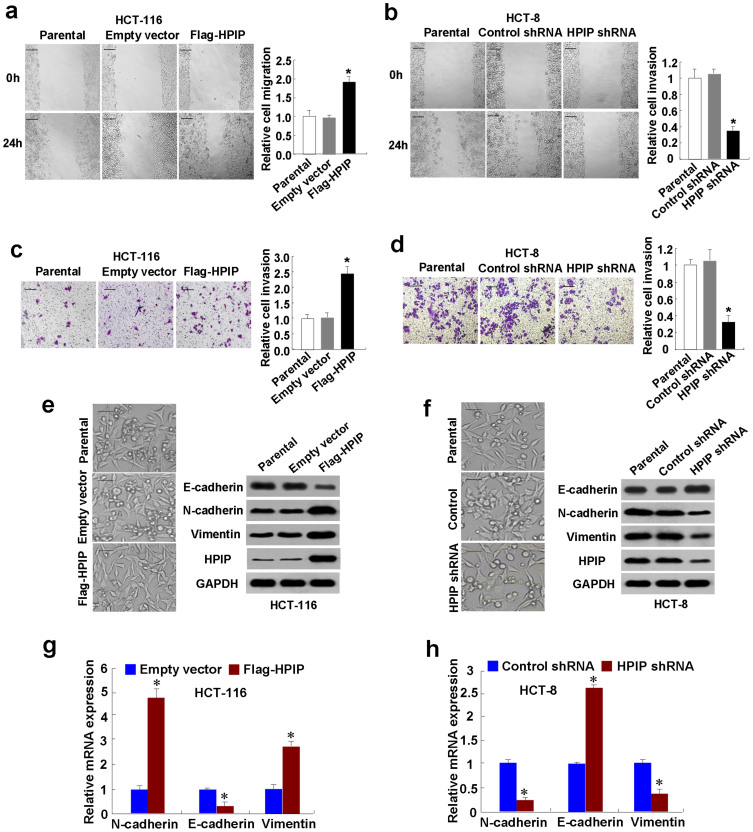
HPIP promotes CRC cell migration and invasion with increased EMT. (A and B) Migration of (A) HPIP-overexpressing HCT-116 cells and (B) HPIP knockdown HCT-8 cells was determined by wound healing assay. The experiments have been repeated three times with similar trends and the image displayed is one of the representative results. (C and D) Cell invasion of (C) HPIP-overexpressing HCT-116 cells and (D) HPIP knockdown HCT-8 cells was assessed by the Matrigel invasion chamber. Invasive cells were fixed and stained with crystal violet. Scale bar: 100 μm. The number of invaded cells in (C) HCT116 or (D) HCT-8 cells was counted. All values shown are mean ± SD of triplicate measurements and have been repeated 3 times with similar results. *p < 0.05 versus empty vector or control shRNA. (E and F) HCT-116 cells (E) were transfected as in (A) and HCT-8 cells (F) were infected as in (B). Morphologic changes are shown in the photographs. Scale bar: 100 μm. Whole cell extracts were used for representative Western blot analysis with the indicated antibodies. (G and H) RT-qPCR analyses for N-cadherin, E-cadherin and Vimentin mRNA expression in HPIP-overexpressing HCT-116 cells (G) and HPIP knockdown HCT-8 cells (H). Data shown are mean ± SD of 3 independent experiments. *p < 0.05, *p < 0.01 versus corresponding empty vector or control shRNA.

To test the effects of HPIP on CRC cell migration and invasion, wound-healing and transwell invasion assays were used. Wound-healing assay demonstrated that overexpression of HPIP in HCT-116 cells increased migration ability (Fig. 5A), while knockdown of HPIP in HCT-8 cells reduced cell motility (Fig. 5B). Transwell invasion assay revealed that overexpression of HPIP in HCT-116 cells enhanced the number of invaded cells (Fig. 5C), whereas knockdown of HPIP in HCT-8 cells reduced the number of invaded cells (Fig. 5D). Consistent with the results of HPIP modulation of CRC cell migration and invasion, overexpression of HPIP promoted epithelial-mesenchymal transition (EMT), which has been shown to play a critical role in cancer cell migration and invasion^7^, with morphologic changes from a polarized epithelial phenotype to an elongated fibroblastoid phenotype, whereas HPIP knockdown repressed EMT (Fig. 5E, 5F). Moreover, HPIP overexpression decreased expression of the epithelial marker E-cadherin and increased that of N-cadherin and Vimentin, two mesenchymal markers (Fig. 5E & Supplementary Fig. 7A–E). Opposite results were obtained in HPIP knockdown cells (Fig. 5F & Supplementary Fig. 7F–J).

To investigate how HPIP regulates EMT markers, we performed RT-qPCR to test the effects of HPIP on the expression of N-cadherin, Vimentin and E-cadherin at mRNA levels. HPIP overexpression enhanced N-cadherin and Vimentin mRNA levels, and decreased E-cadherin mRNA level (Fig. 5G). Opposite results were obtained in HPIP knockdown cells (Fig. 5H). Next, we used promoter luciferase reporter assay to further confirm if HPIP regulates the transcription of EMT markers. We found that HPIP increased the promoter activities of N-cadherin and Vimentin, and reduced that of E-cadherin (Supplementary Fig. 3D).

### HPIP increases CRC cell proliferation, migration and EMT through activation of MAPK and AKT

**Figure 6:**
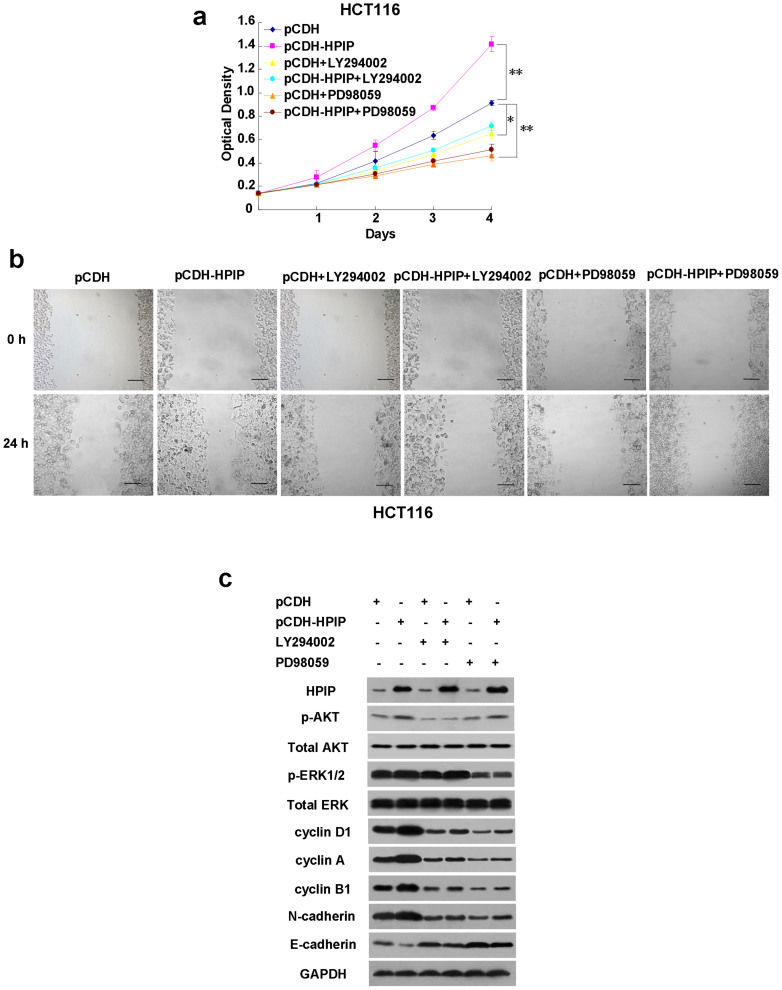
HPIP increases CRC cell proliferation, migration and invasion through activation of MAPK and AKT. (A) HCT-116 cells were infected with lentivirus expressing HPIP (pCDH-HPIP) or empty vector (pCDH), and were treated for 24 h with 10 μM LY294002 or 10 μM PD98059. After 24 h, the culture medium was changed with fresh drug-free medium, and the cells were grown for the indicated times. Cell number was determined by CCK-8 assay. (B) Wound healing assays for HCT-116 cells infected as in (A) and treated with LY294002 or PD98059 for 24 h. Scale bar: 100 μm. (C) Representative Western blot analysis of HCT-116 cells infected and treated as in (B). p-AKT: phosphorylated AKT, p-ERK1/2: phosphorylated ERK1/2. All values shown are mean ± SD of triplicate measurements and have been repeated 3 times with similar results (*p < 0.05, **p < 0.01).

Mitogen-activated protein kinase (MAPK)/extracellular signal-regulated kinase 1/2 (ERK1/2) and protein kinase B/AKT are activated by phosphorylation on their specific sites and are critical players in tumorigenesis and progression. Since HPIP has been shown to activate MAPK/ERK1/2 and AKT in breast cancer and liver cancer cells^5^,^8^, we investigated whether activation of MAPK/ERK1/2 and AKT is responsible for HPIP modulation of CRC cell proliferation, migration and EMT. We treated HPIP-overexpressing HCT-116 and SW480 cells with PD98059 and LY294002, which are MAPK/ERK1/2 and PI3K/AKT inhibitors, respectively. As expected, HPIP overexpression in HCT116 and SW480 cells promoted cell proliferation and migration, and increased the expression of EMT markers, accompanied by elevated levels of AKT and ERK1/2 phosphorylation, increased expression of cyclin D1, cyclin A and cyclin B1, and the EMT markers N-cadherin and Vimentin, and decreased expression of the EMT marker E-cadherin (Fig. 6 & Supplementary. Fig. 4). Treatment with LY294002 or PD98059 blocked the phosphorylation of AKT and ERK1/2, respectively (Fig. 6C & Supplementary. Fig. 4C & Supplementary Fig. 8A–E). Importantly, treatment with LY294002 or PD98059 greatly alleviated the ability of HPIP to regulate CRC cell proliferation and migration as well as the expression of the cell cycle regulators and the EMT markers (Fig. 6C & Supplementary. Fig. 4C & Supplementary Fig. 8F–K). These results suggest that activation of MAPK and AKT is responsible for HPIP modulation of CRC cell proliferation, migration and EMT.

### Knockdown of HPIP suppresses CRC cell growth in nude mice

**Figure 7:**
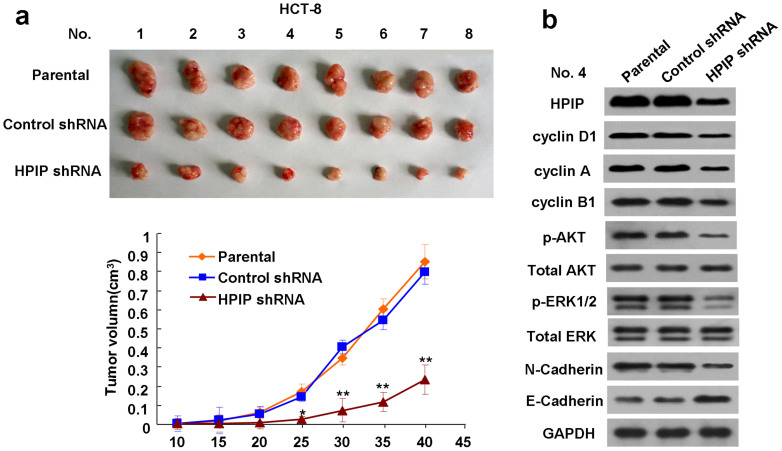
Knockdown of HPIP suppresses CRC cell growth in nude mice. (A) HCT-8 cells stably infected with HPIP shRNA or control shRNA or parental HCT-8 cells were injected into nude mice. At the indicated times, tumors were measured with Vernier calipers (mean ± SD; n = 5). **p < 0.01 versus corresponding control shRNA. (B) Immunoblot analysis of representative excised tumor from (A).

Finally, the effect of HPIP knockdown on CRC cell growth in nude mice was investigated. HCT-8 cells stably infected with HPIP shRNA lentivirus or empty vector, or parental HCT-8 cells were injected subcutaneously in the dorsal of each nude mouse. Compared with the control groups, knockdown of HPIP significantly suppressed CRC growth in nude mouse (Fig. 7A). As expected, the HCT-8 tumors in mice inoculated with HPIP shRNA showed decreased expression of HPIP, cyclin D1, cyclin A, cyclin B1, N-cadherin, and phosphorylation of AKT and ERK1/2, and increased expression of E-cadherin (Fig. 7B).

## Discussion

HPIP can promote proliferation and migration/invasion of breast, liver and brain cancer cells^5^,^6^,^7^,^8^,^9^. However, the role of HPIP in colorectal cancer is unknown. Our present work suggests critical function of HPIP in colorectal cancer. First, HPIP is upregulated in colorectal cancer patients and predicts bad clinical outcome. Second, HPIP promotes cell growth by inhibiting apoptosis and activation of cell cycle progression, accompanied by changes in expression of apoptosis and cell cycle regulators. Third, HPIP promotes cell migration and invasion with increased EMT. Mechanistically, HPIP increases CRC cell proliferation and migration through activation of MAPK/ERK1/2 and AKT. Finally, knockdown of HPIP can inhibit CRC cell growth in nude mice. These findings indicate that HPIP may play an important role in the development and progression of colorectal cancer.

Although HPIP has been shown to regulate cell proliferation, whether HPIP has a role in regulation of apoptosis remains unclear. We found that expression of HPIP inhibits apoptotic processes along with increased ratio of BCL2/BAX and decreased PIG3 expression as well as reduced caspase-3-mediated PARP cleavage. The BCL2 family of proteins represents key regulators of apoptosis and can be classified into two functionally distinct groups: anti- and pro-apoptotic proteins^10^. BCL2 is a critical anti-apoptotic BCL2 family member, and BAX, acting as a promoter of apoptosis, belongs to pro-apoptotic family members. The apoptosis are more dependent on the balance between BCL2 and BAX than on BCL2 quantity alone^11^. Among caspases involved in apoptosis, caspase-3 is a critical executioner of apoptosis. caspase-3 is crucial for apoptotic chromatin condensation and DNA fragmentation^12^. PARP is the substrate of caspase-3. Caspase-3-mediated PARP cleavage indicates cell apoptosis, so PARP is known as "death substrate". Furthermore, triggering of the caspase cascade is paralleled by transcriptional activation of the PIG3 gene^13^,^14^,^15^,^16^. Interestingly, based on apoptosis, HPIP reduces sensitivity of CRC cells to Oxaplatin, a chemotherapy drug used for treatment of CRC. Whether HPIP plays a role in Oxaplatin resistance remains to be investigated.

Abnormal regulation of cell cycle is a remarkable characteristic of cancer cells^17^. Studies have demonstrated that many small molecules can activate cell cycle checkpoints and induce cell cycle arrest, which allow cells to repair defects. G2/M cell cycle checkpoint control is important for insuring cells not to initiate mitosis before they have a chance to repair damaged DNA after replication^18^,^19^,^20^,^21^. The cyclin B-CDK1 complex plays an essential role in the regulation of the G2/M checkpoint. Transition from G1 to S phase requires the activation of assembly of cyclin D1-CDK4/6 and cyclin A-CDK1. HPIP has been shown to regulate G2/M checkpoint in liver cancer cells^8^. In this study, we show that, in CRC cells, HPIP modulates both the G1/S and the G2/M transitions, along with increased expression of cyclin D1, cyclin A, and cyclin B, suggesting that HPIP is an important cell cycle regulator. The discrepancy of HPIP-mediated cell cycle control between liver cancer and CRC might be due to different genetic background and different signaling network between the two cancers. For instance, the breast cancer susceptibility gene BRCA1 was shown to be involved in all phases of the cell cycle and modulate orderly events during cell cycle progression^22^. However, in different cell lines, BRCA1 exhibits different cell cycle control patterns. Aprelikova et al. demonstrated that BRCA1 induced accumulation of human osteosarcoma U2OS cells in G1 phase of cell cycle^23^. Such effect was not observed in HBL100 cells (normal breast epithelial cells immortalized with simian virus 40). U2OS cells harbor wild-type retinoblastoma tumor suppressor (RB), whereas HBL100 cells express inactivated RB because of the presence of simian virus 40. They further found that only cells carrying wild-type RB were sensitive to BRCA1-induced G1 arrest, while RB−/− cells were not. The mechanisms by which HPIP differentially regulates cell cycle in liver cancer and CRC cells need to be investigated. In addition, HPIP knockdown had more marked effects on liver xenograft tumor growth^8^ than on CRC tumor growth. The underlying mechanisms might be similar to those underlying distinct HPIP-mediated cell cycle control between liver cancer and CRC cells. For example, Ret is an oncogene in thyroid cancer, while it is a tumor suppressor gene in colorectal cancer^23^,^24^.

The epidermal growth factor receptor (EGFR) pathway plays a critical role in CRC development and progression^25^,^26^. The binding of EGF or other ligands to EGFR initiates a mitogenic signaling cascade through two main axes, the KRAS-RAF-MAPK/ERK1/2 pathway and the PI3K-AKT pathway. Both ERK1/2 and AKT are frequently activated in CRC and are critical for CRC cell growth^27^. ERK1/2 and AKT also induce EMT, an important step toward cancer cell migration, invasion and metastasis^28^,^29^,^30^,^31^. Our study shows that inhibition of ERK1/2 and AKT by PD98059 and LY294002 in HPIP-overexpressing CRC cells attenuates the ability of HPIP to promote CRC cell proliferation and migration and to regulate the expression of the important cell cycle regulators cyclin D1, cyclin A and cyclin B1 as well as the EMT regulators E-cadherin and N-cadherin, suggesting that activation of ERK1/2 and AKT is critical for HPIP modulation of the proliferation and migration of CRC cells and of the expression of cell cycle and EMT regulators. Since HPIP is overexpressed in CRC patients and promotes tumor growth in vivo, inhibition of ERK1/2 and AKT or inhibition of HPIP may be useful strategies for the treatment of HPIP-overexpressing CRC patients.

## Methods

### Immunohistochemistry (IHC)

Colorectal cancer samples and adjacent noncancerous tissues were obtained from the China PLA second Artilery General Hospital with the informed consent of patients. Before surgical therapy, none of the patients had received neoadjuvant chemotherapy, radiation therapy or immunotherapy. Study protocol was approved by the Ethics Committee of Chinese PLA Second Artilery General Hospital and all experimental methods were carried out in accordance with approved guidelines of academy of military medical sciences. The IHC procedure was performed as described previously^32^. Briefly, the antigens were retrieved by microwave treatment and incubated with rabbit anti-HPIP antibody (Proteintech, Chicago, USA) at a dilution of 1/100. Bound primary antibodies were detected by the addition of biotinylated goat anti-rabbit secondary antibody and streptavidin-horseradish peroxidase (Zymed Laboratories). The color was developed with 3, 3′-diaminobenzidine. The samples were counterstained using hematoxylin. For negative controls, normal rabbit IgG (Santa Cruz Biotechnology) or phosphate-buffered saline was substituted for the primary antibody. All of IHC staining was assessed by two pathologists blinded to the origination of the specimen. The widely accepted German semi-quantitative scoring system in considering the staining intensity and area extent was used: 0, no staining; 1, weak staining; 2, moderate staining; and 3, strong staining; In addition, the percentage of staining was given a score of 0 (<5%), 1 (5%–25%), 2 (25%–50%), 3 (51%–75%), 4 (>75%). Two scores mentioned above were multiplied as the final score. For HPIP, we defined 0 score as negative and 1–12 as positive.

### Plasmids, cell lines and reagents

FLAG-tagged HPIP expression vector was constructed by inserting PCR amplified HPIP fragment into a pcDNA3 vector (Invitrogen) linked with FLAG tag at the amino terminus. Lovo, Caco2, HT-29 and LS174 CRC cell lines were purchased from the American Type Culture Collection (Manassas, VA, USA). HCT-116 and HCT-8 CRC cell lines were kind gifts from Professor Zhiwei Sun at Beijing Institute of Biotechnology. SW480 CRC cell line was a gift from Professor Chengchao Shou at Peking University Cancer Hospital. Stable cell lines overexpressing HPIP were established by lentiviral transduction using pCDH plasmid (System Biosciences). Stable HPIP knockdown cell lines were established by cloning HPIP short hairpin RNA (shRNA) fragment into the lentiviral vector pSIH-H1-Puro (System Biosciences). The sequence of HPIP shRNA has been described previously^6^. Lentivirus was generated by transfection of the 293T producer cell line with the lentiviral vector and packing vector mix (System Biosciences). Lentivirus was collected 48 h later, which was used to infect CRC cells. Stable cell lines expressing HPIP or HPIP shRNA were selected with puromycin 48 h after infection. Pooled clones were screened by immunoblot with anti-HPIP. Similar results were obtained with individual clones.

Anti-cyclin D1, anti-ERK1/2, anti-AKT, anti-phos-AKT(T308), and anti-phos-ERK1/2(T202/Y204) were purchased from Santa Cruz Biotechnology; Anti-E-cadherin and anti-N-cadherin were from BD Biosciences; Anti-Vimentin, anti-caspase-3, and anti-PARP were obtained from Cell Signaling Technology; Anti-HPIP was purchased from Proteintech; Anti-BAX and anti-PIG-3 were purchased from Abcam.

### Transient Transfections

Human colorectal cancer cell lines were routinely cultured in RPMI 1640 medium supplemented with 10% fetal bovine serum at 37°C in humidified atmosphere of 5% CO_2_ in air. For transfection, cells were seeded in 24-well or 6-well plates in triplicate containing DMEM supplemented with 10% fetal bovine serum. The cells were transfected with the indicated plasmids using Vigofect according to the manufacturer's protocol (Vigorous Biotechnology).

### Cell growth and colony formation assays

Anchorage-dependent cell growth was evaluated by the CCK-8 Kit (Dojindo Laboratories) according to the manufacturer's instructions. For colony formation assay, transfected cells were seeded in 6-well plates at 2000 cells per well. Two weeks later, colonies were fixed with 4% paraformaldehyde and stained with crystal violet for 30 min. The number of colonies with diameters of more than 1.5 mm was counted.

### Cell cycle analysis

Cell cycle analysis was carried out using flow cytometry as described previously^33^. Briefly, cells were fixed in 70% ethanol for more than 18 h, washed with PBS, and incubated with RNase A (0.2 mg/mL) in PBS. Propidium Iodide was then added to the cell suspension. Samples were analyzed by a FACScalibur Flow Cytometer (Becton Dickinson).

### Apoptosis and flow cytometry analysis

Stable HPIP-overexpressing, HPIP- knock down or empty vector control cells (1 × 10^6^ cells) were cultured in 60 mm dishes for 48 h and harvested. The cells were labeled with propidium iodide and Annexin V according to the manufacturer's instructions (BD Biosciences). A minimum of 10,000 events for each sample were collected and analyzed using a FACScalibur Flow Cytometer (Becton Dickinson).

### Quantitative reverse-transcription PCR (RT-qPCR)

Total RNA was extracted from cultured cells and reverse-transcribed to cDNA using the RNeasy Mini kit (Qiagen) according to the manufacturer's protocol. Expression of mRNAs was determined using SYBR Premix Ex Taq Master Mix (2×) (Takara). The relative expression was calculated by the comparative Ct method. The sequences of the primers used for RT-qPCR analysis are presented in Supplementary Table 1.

### Luciferase reporter assay

Cells seeded into 24-well plates were co-transfected with HPIP and luciferase reporter constructs containing different promoter regions (for E-Cadherin promoter, −1201 to −10 bp; for N-Cadherin promoter, −865 to +10 bp, for Vimentin promoter, −1006 to +5 bp, for cyclin A promoter, −783 to 0 bp, for cyclin D1 promoter, −1392 to 0 bp). Cells were harvested and analyzed for luciferase and β-galactosidase activities as previously described^9^. All transfection experiments were performed in triplicates and reproduced at least three times.

### Cycloheximide (CHX) chase assay

Transfected cells were treated with 20 μg/ml cycloheximide for different time periods. Cell lysates were analyzed by immunoblotting with indicated antibodies.

### Cell migration and invasion assays

Wound healing assays were performed to assess cell migration. Briefly, transfected cells cultured in 6-well plates as confluent monolayers were mechanically scratched using a 1-ml pipette tip to create the wound. Cells were washed with PBS to remove the debris and were cultured for 24 h to allow wound healing. Cell invasion was determined with Matrigel (BD Biosciences) coated on the upper surface of the transwell chamber (Corning). Twenty-four hours later, cells invaded through the Matrigel membrane were fixed with 4% paraformaldehyde and stained with crystal violet. The number of invaded cells was counted in 5 randomly selected microscopic fields and photographed.

### In vivo tumor growth

Animal studies were approved by the Institutional Animal Care Committee of Beijing Institute of Biotechnology. HCT-8 cells stably infected with pSIH control vector or pSIH-HPIP shRNA were injected subcutaneously in the dorsal of each animal (6-week-old male nude mice) (n = 5). Tumor size was measured at indicated times using calipers. Tumor volume was estimated according to the following formula: volume = (longest diameter × shortest diameter^2^)/2. The mice were sacrificed 3 weeks after the first intratumoral injection, and tumors were excised, measured, and weighed.

### Statistical analysis

All in vitro experiments were performed in triplicate and repeated 3 times. The difference of HPIP expression between colorectal cancers and normal tissues was assessed by Mann-Whitney *U*-test. Estimation of disease-free survival and overall survival was performed using the Kaplan-Meier method, and differences between survival curves were examined with the log-rank test. Statistical significance in cell proliferation, apoptosis and invasion assays among constructs was determined by two-tailed Student's t-test. The SPSS 17.0 statistical software package was used to perform the statistical analyses. *p*<0.05 was considered statistically significant.

## Author Contributions

Q.Y. and X.X. conceived the project, designed the experiments and analyzed the data. K.Z. and S.S. supervised the study. Y.F. and X.X. performed the experiments, supported by Y.Z., J.D., Y.W., X.Z., Z.W., L.K., Y.L. and L.Z., Q.Y., Y.F. and X.X. wrote the manuscript. All authors read and approved the final manuscript.

## Supplementary Material

Supplementary Information Supplementary figure and table
