# Supplementary material for: HPIP is upregulated in colorectal cancer and regulates colorectal cancer cell proliferation, apoptosis and invasion
Source: Sci Rep. 2015 Mar 24;5:9429. doi: 10.1038/srep09429 (PMC4371107; doi:10.1038/srep09429)
Supplement: Supplementary Information — Supplementary figure and table [file srep09429-s1.pdf]

Supplementary Information

HPIP is upregulated in colorectal cancer and regulates colorectal cancer cell proliferation, apoptosis and invasion

Yingying Feng, Xiaojie Xu, Yunjing Zhang, Jianhua Ding, Yonggang Wang, Xiaopeng Zhang, Zhe Wu, Lei Kang, Yingchun Liang, LiYing Zhou, Santai Song, Ke Zhao & Qinong Ye

— Supplementary Figure S1

— Supplementary Figure S2

— Supplementary Figure S3

— Supplementary Figure S4

— Supplementary Figure S5

— Supplementary Figure S6

— Supplementary Figure S7

— Supplementary Figure S8

— Supplementary Table S1

Figure S1

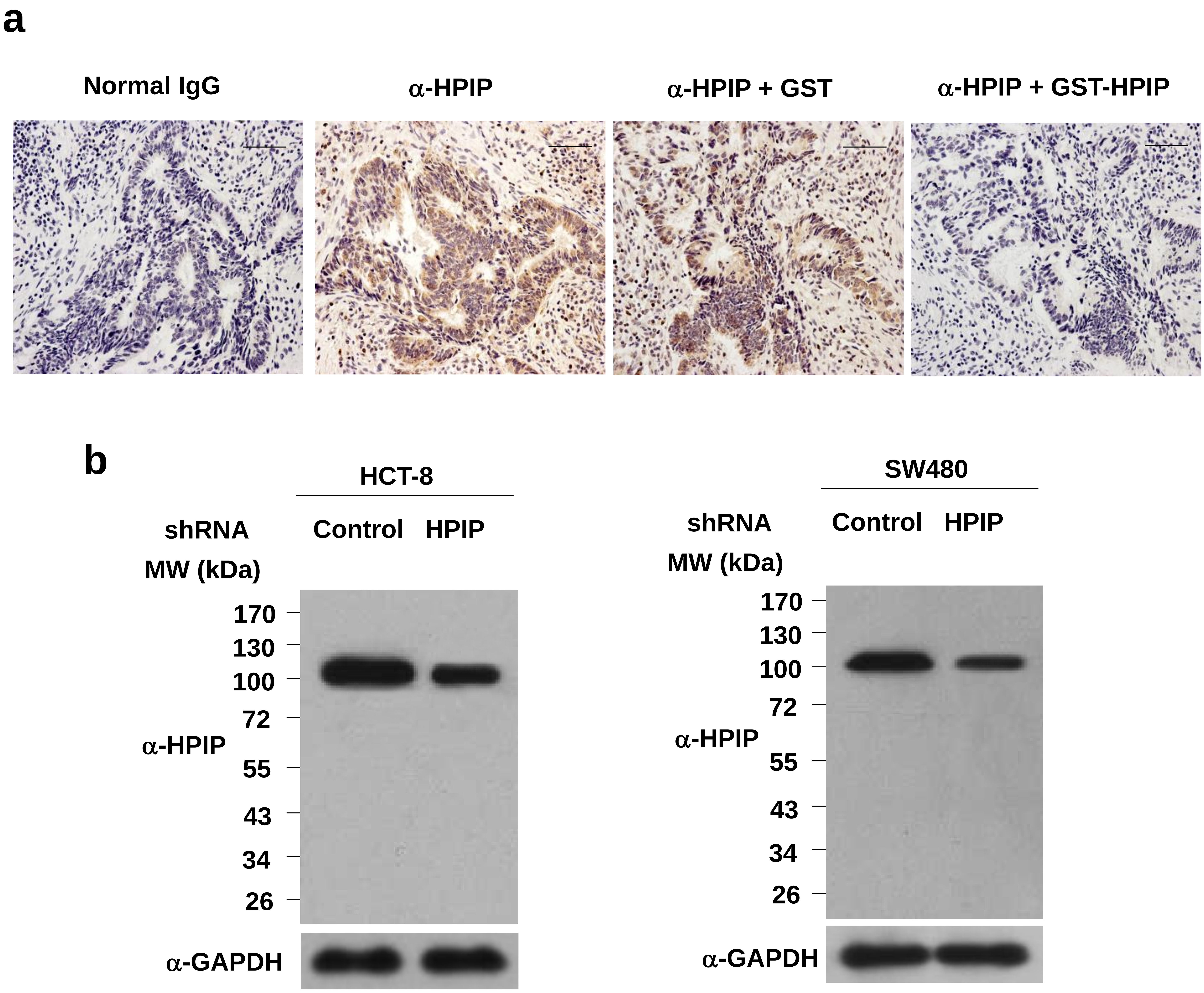

Supplementary Figure 1. Validation of antibody specificity to HPIP.
(**a**) Immunohistochemical staining of CRC samples incubated with normal IgG or anti-HPIP. To validate antibody specificity, the anti-HPIP was pre-incubated with recombinant GST-HPIP protein or GST for 1 h prior to applying to tissue. Original magnification, ×20. Scale bar, 100 μm. **(b)** Immunoblot analysis of lysates from HCT-8 (left panel) or SW480 (right panel) cells infected with control shRNA or HPIP shRNA using antibodies specific for anti-HPIP. MW, molecular weight.

Figure S2

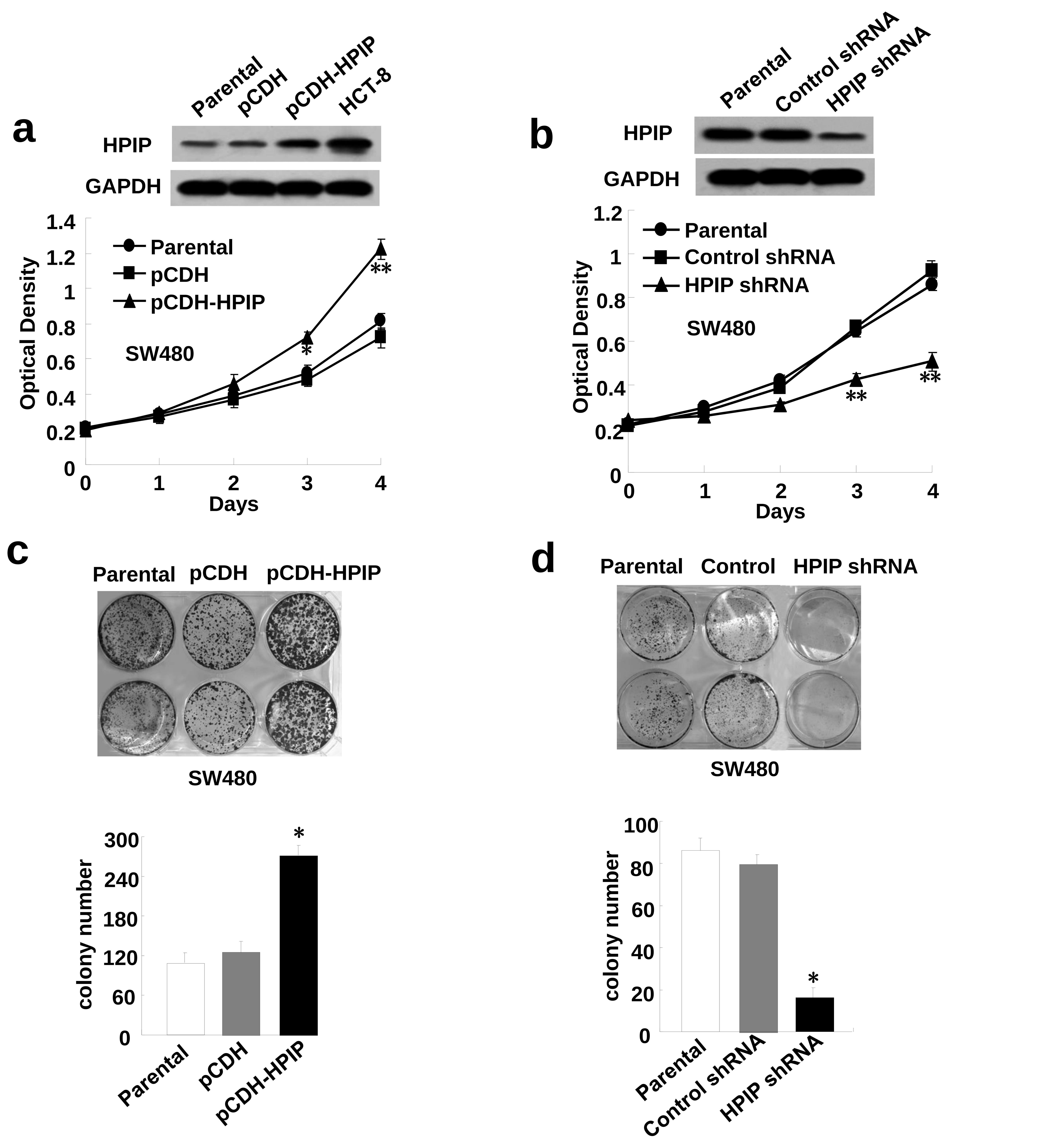

Supplementary Figure 2. HPIP regulates SW480 cell proliferation. (a & b) SW480 cells infected with lentivirus expressing **(a)** HPIP (pCDH-HPIP) or **(b)** HPIP shRNA were cultured for the indicated times. Cell number was determined by CCK-8 assay. Parental: parental cells. *$p < 0.05$ versus empty vector or control shRNA, **$p < 0.01$ versus empty vector or control shRNA or parental cells. Immunoblotting showed the expression of HPIP. HCT-8 cells were used for comparison of HPIP expression levels in different groups. **(c & d)** Colony formation assays for SW480 cells infected as in (a) and (b) (*$p < 0.05$). All values shown are mean ± SD of triplicate (a and b) or duplicate (c and d) measurements and have been repeated 3 times with similar results.

Figure S3

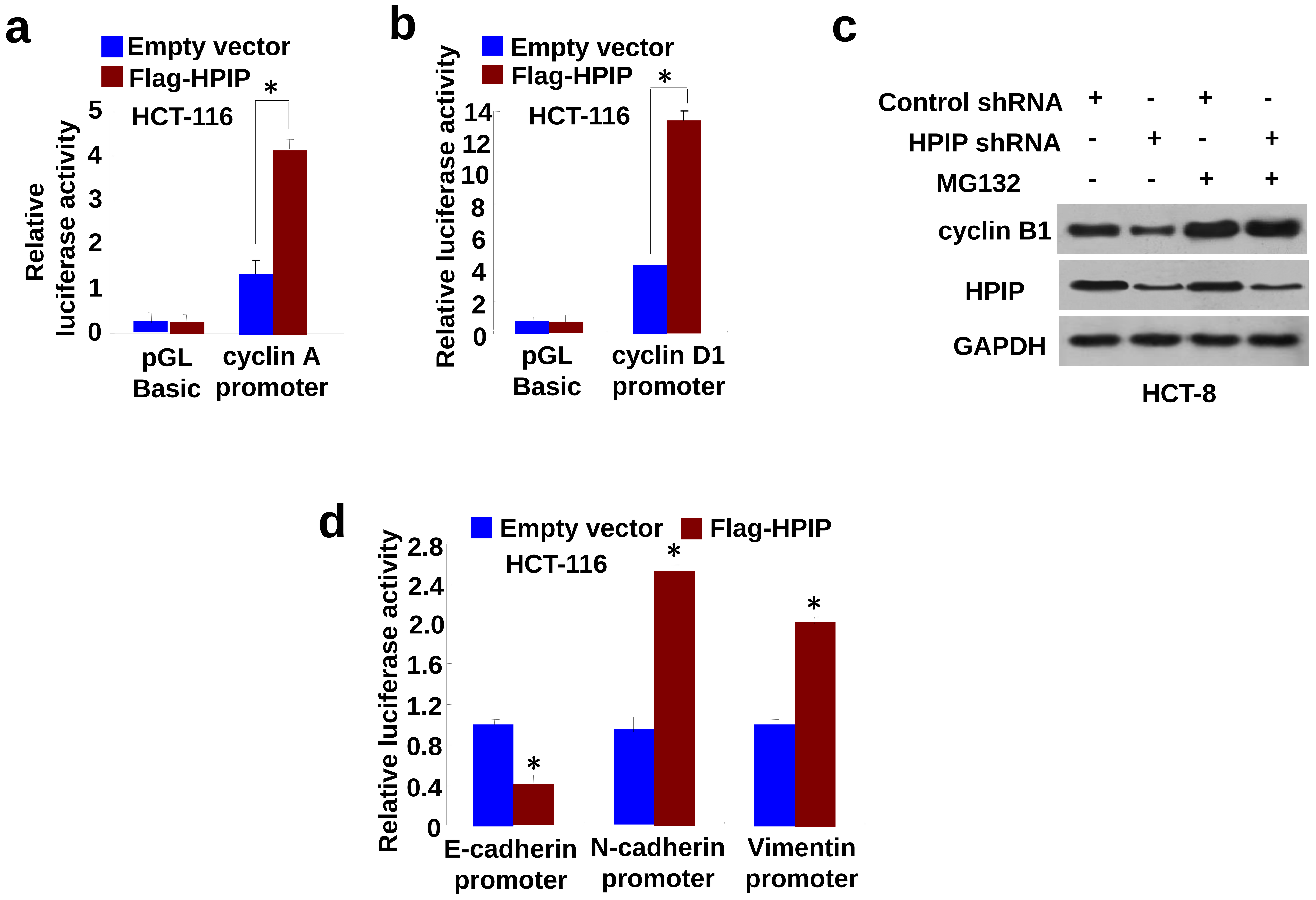

Supplementary Figure 3. HPIP regulates the expression of cyclins and the EMT markers. (a & b) Luciferase reporter assays in HCT-116 cells transfected with FLAG-tagged HPIP and the cyclin A-Luc or cyclin D1-Luc reporter. **(c)** Immunoblot analysis of HCT-8 cells stably transfected with HPIP shRNA or control shRNA and treated with the proteasome inhibitor MG132 (10 μM). **(d)** Luciferase reporter assays in HCT-116 cells transfected with FLAG-tagged HPIP and the E-cadherin-Luc, N-cadherin-Luc or Vimentin-Luc reporter. Data shown are mean ± SD of triplicate measurements that have been repeated 3 times with similar results (*$P < 0.05$ vs corresponding control).

Figure S4

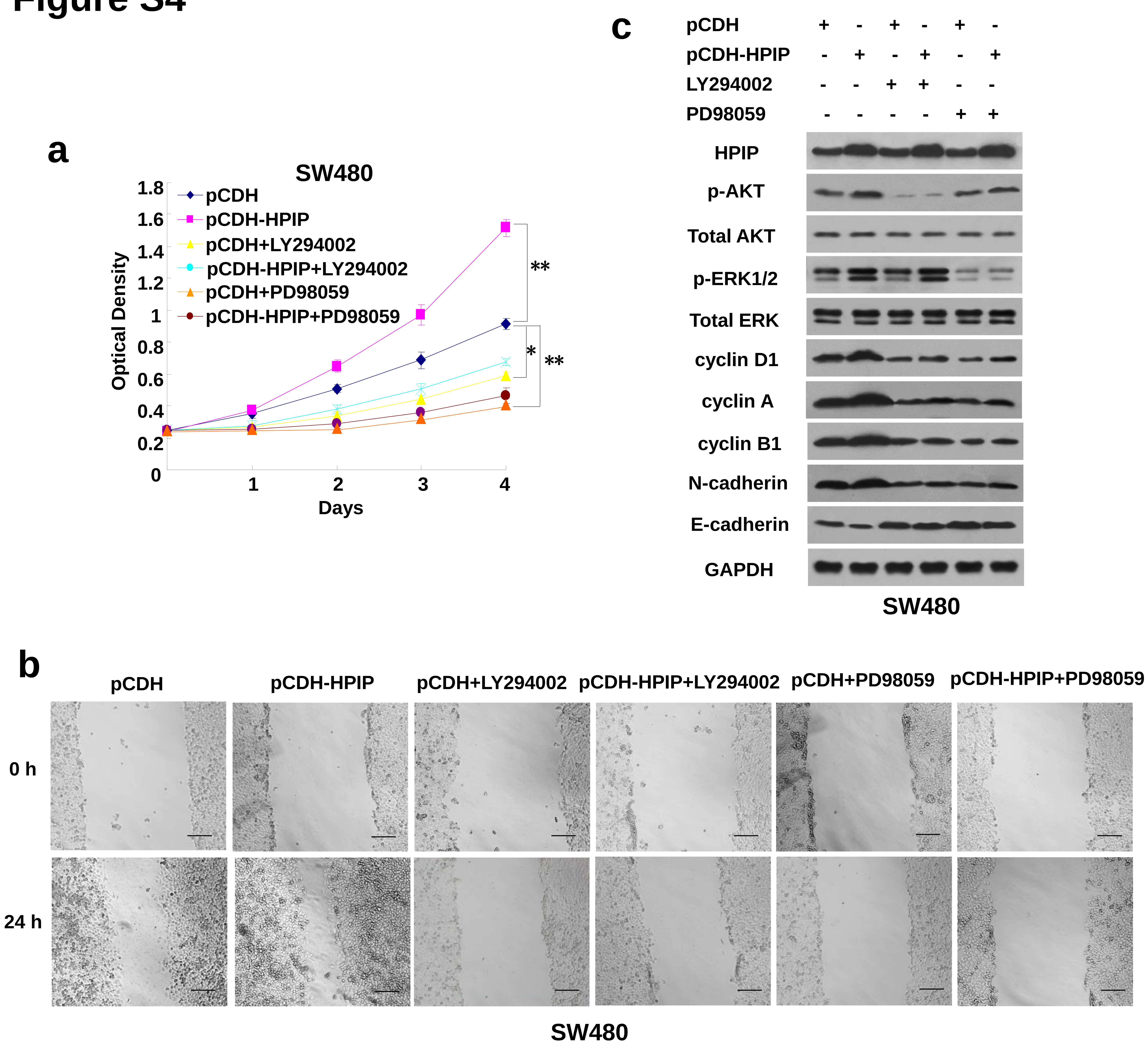

Supplementary Figure S4. HPIP increases SW480 cell proliferation and migration through activation of MAPK and AKT. (a) Cell proliferation assay for SW480 cells infected with lentivirus expressing HPIP (pCDH-HPIP) or empty vector (pCDH), and treated and analyzed as in Figure 6a. **(b)** Wound healing assays for SW480 cells infected and treated as in Figure 6B. Scale bar: 100 μm. **(c)** Western blot analysis of SW480 cells infected and treated as in (a). All values shown are mean $\pm$ SD of triplicate measurements and have been repeated 3 times with similar results (*p < 0.05, **p < 0.01).

Figure S5

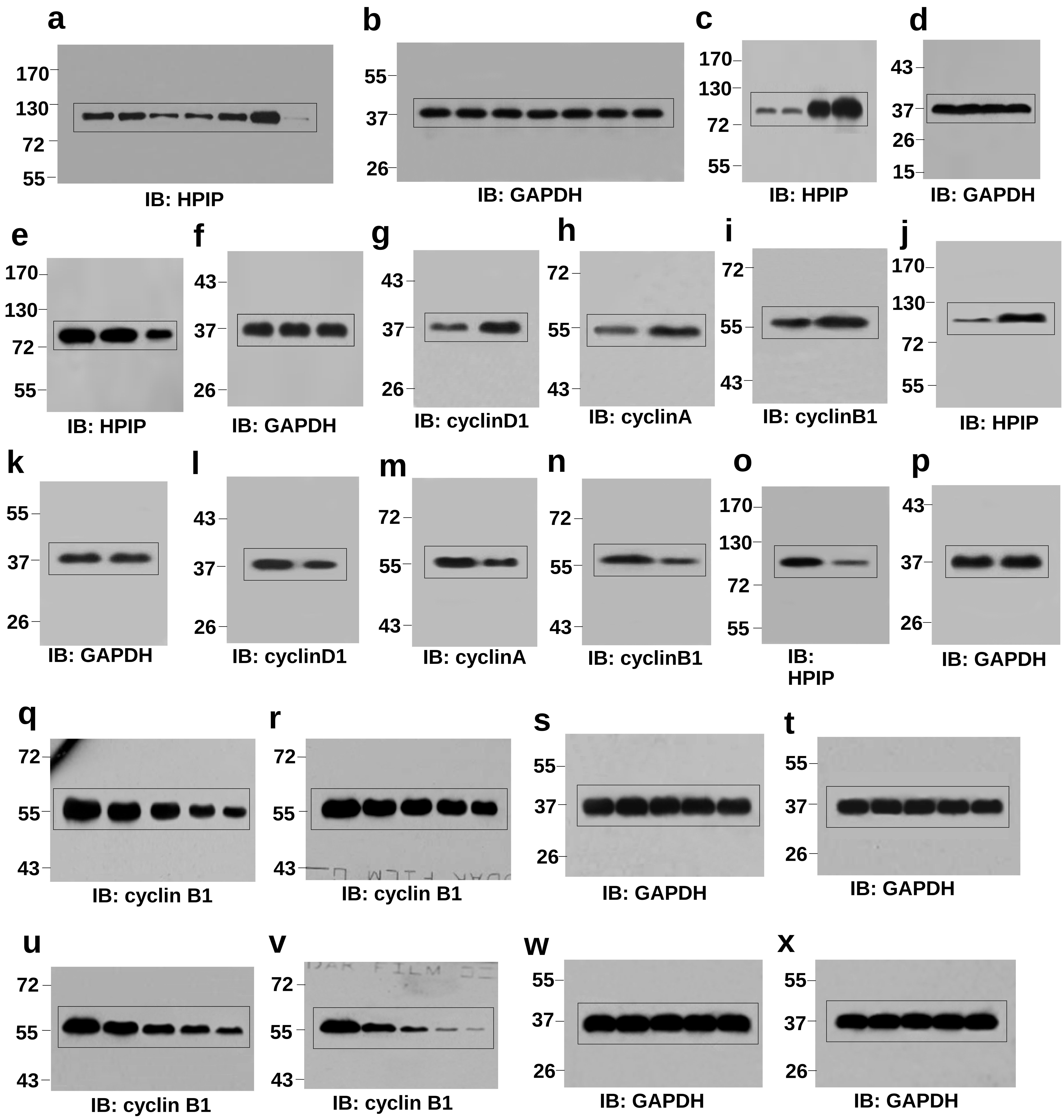

Supplementary Figure S5. Full-length gels and blots for key data in the main figures. (a&b) Full length of gel for Western-blot images of Figure 2A. **(c&d)** Full length of gel for Western-blot images of Figure 2B. **(e&f)** Full length of gel for Western-blot images of Figure 2C. **(g-k)** Full length of gel for Western-blot images of Figure 3C. **(l-p)** Full length of gel for Western-blot images of Figure 3D. **(q-t)** Full length of gel for Western-blot images of Figure 3G. Molecular weight is shown at the left panel (kDa). **(u-x)** Full length of gel for Western-blot images of Figure 3H. Molecular weight is shown at the left panel (kDa).

Figure S6

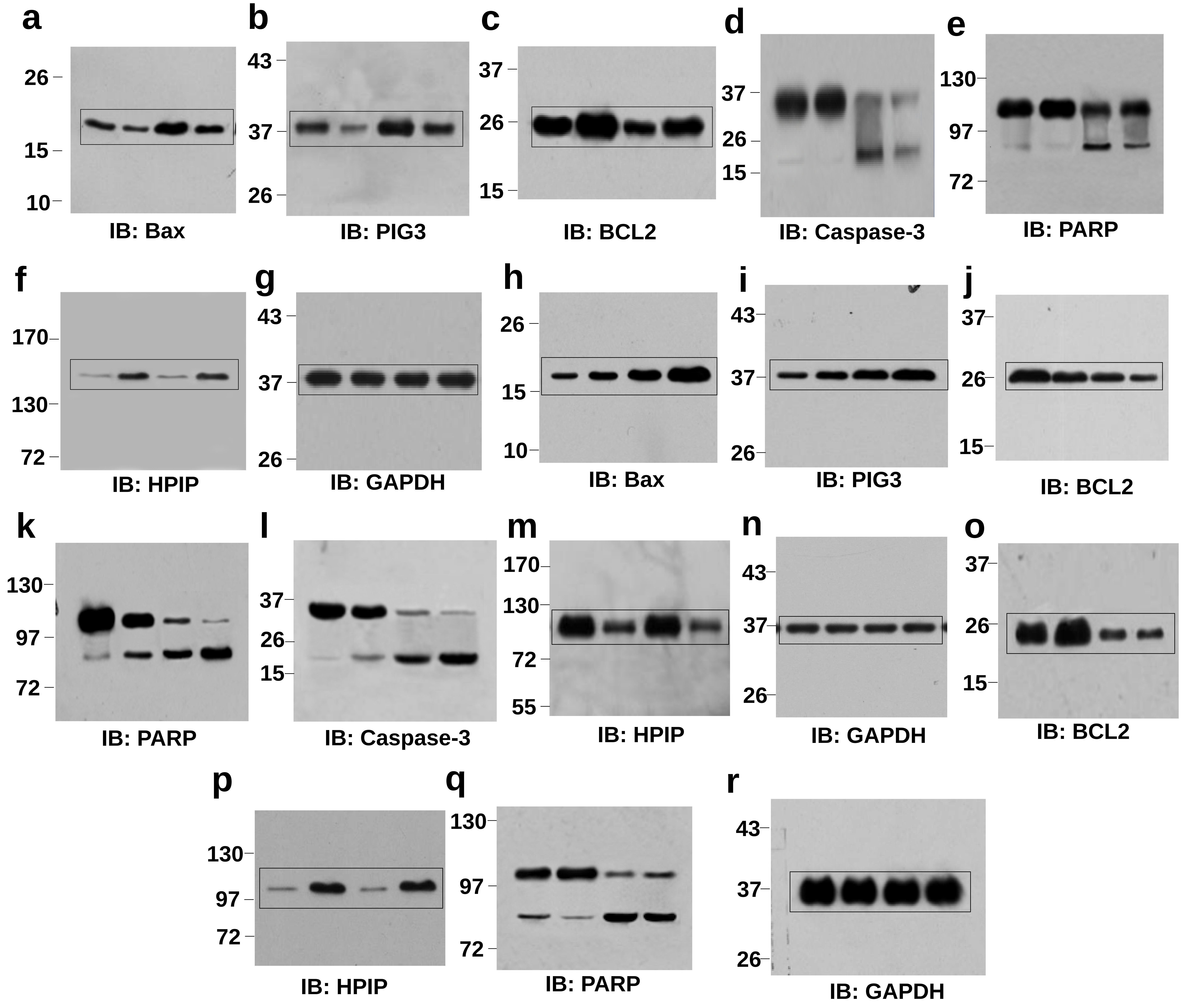

Supplementary Figure S6. Full-length gels and blots for key data in the main figures. (a-g) Full length of gel for Western-blot images of Figure 4C. **(h-n)** Full length of gel for Western-blot images of Figure 4D. **(o-r)** Full length of gel for Western-blot images of Figure 4E. Molecular weight is shown at the left panel (kDa).

Figure S7

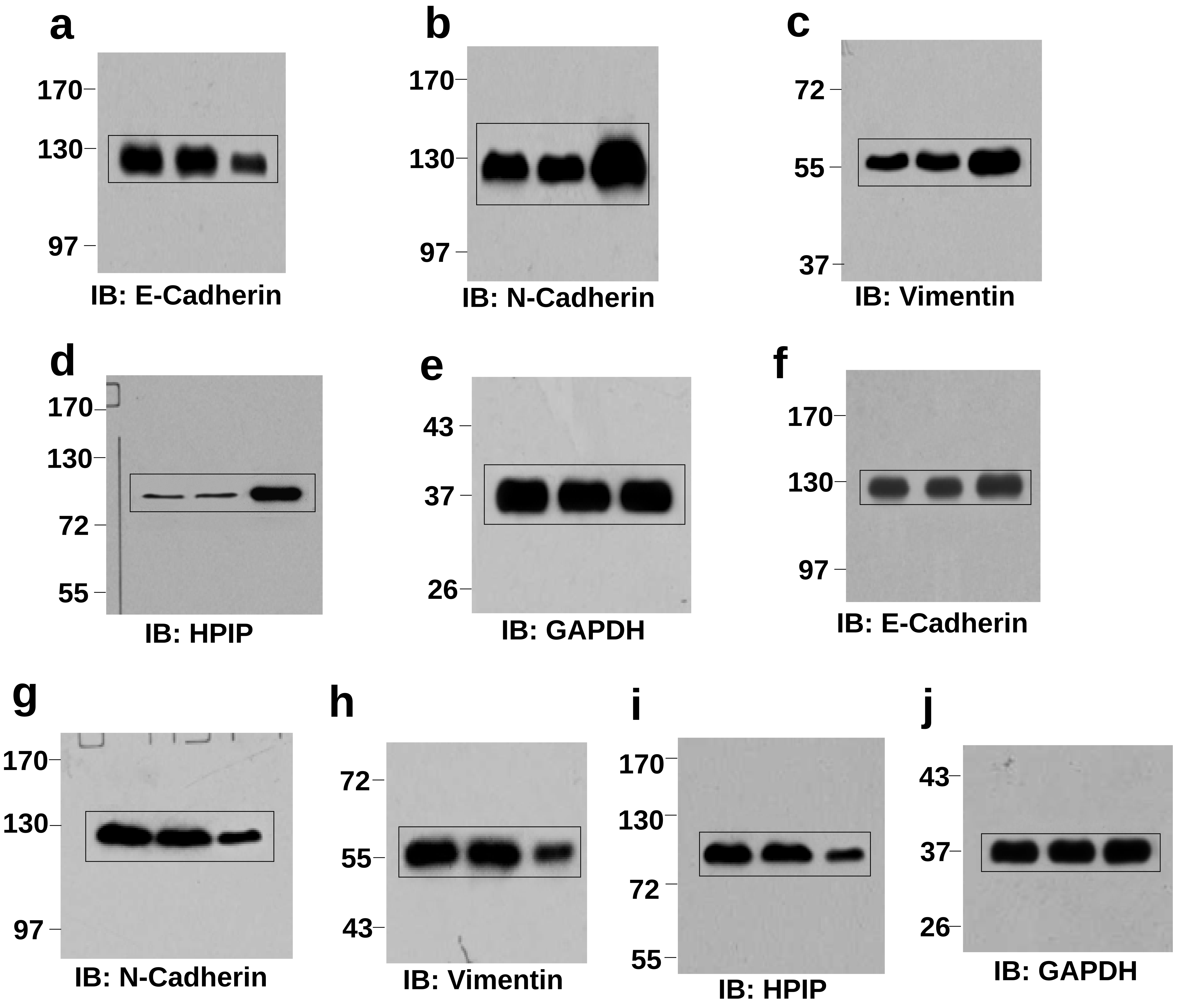

Supplementary Figure S7. Full-length gels and blots for key data in the main figures. (a-e) Full length of gel for Western-blot images of Figure 5E. **(f-j)** Full length of gel for Western-blot images of Figure 5F. Molecular weight is shown at the left panel (kDa).

Figure S8

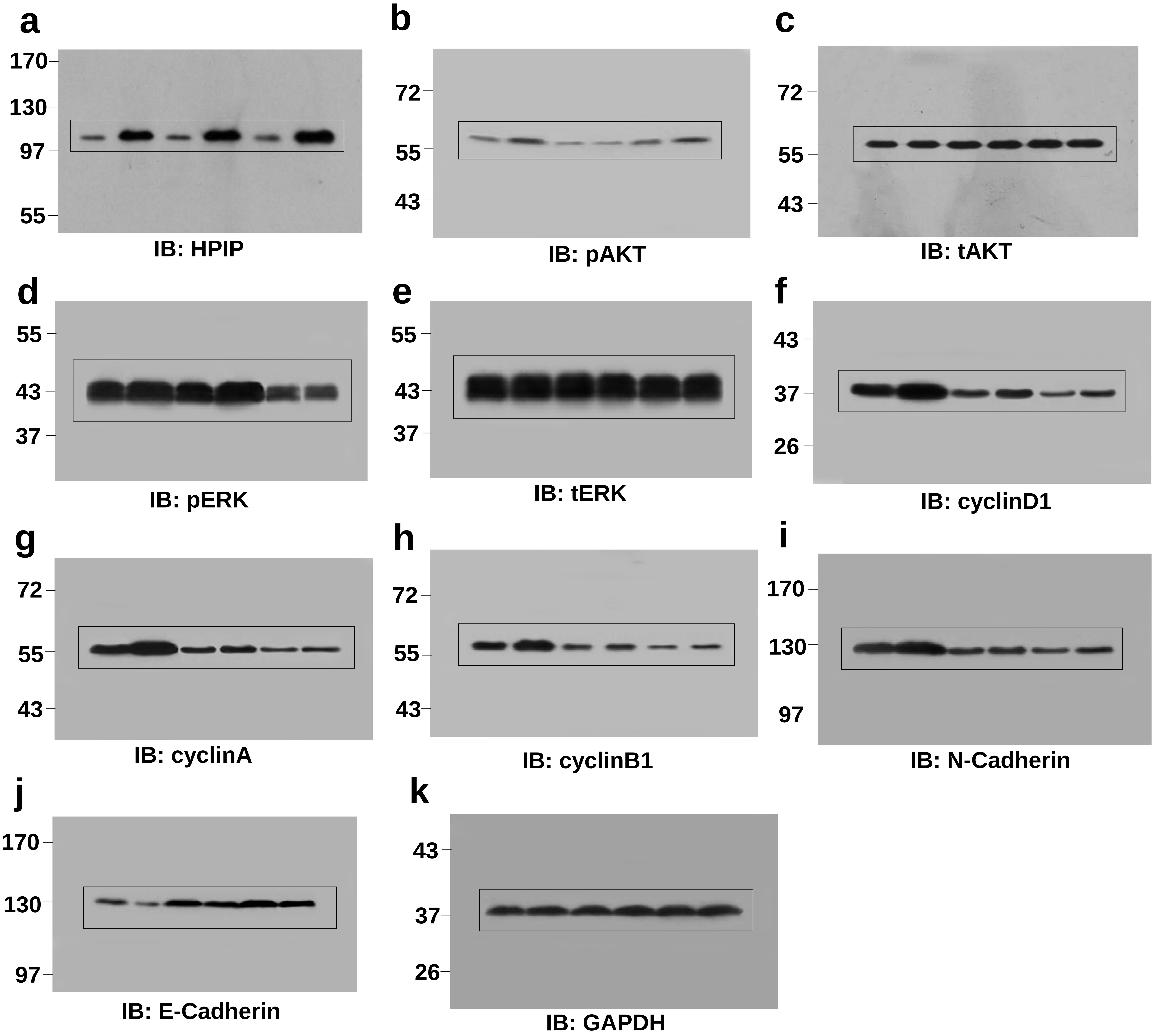

Supplementary Figure S8. Full-length gels and blots for key data in the main figures. (a-k) Full length of gel for Western-blot images of Figure 6C. Molecular weight is shown at the left panel (kDa).

Supplementary Table S1. Primers used for real-time RT-PCR.

Gene	Forward (5'→3')	Reverse (5'→3')
cyclin A	CATACCTCAAGTATTTGCCATC	GGTCCATGAGACAAGGCTTAAG
cyclin D1	CCGCCTCACACGCTTCCTCTC	TCCTCCTCGGCGGCCTTGGGG
cyclin B1	GTCGGCCTCTACCTTTGCACTTCCTTC	GAGTTGGTGTCCATTCACCATTATCCAG
E-cadherin	CCCATCAGCTGCCCAGAAAATGAA	CTGTCACCTTCAGCCATCCTGTTT
N-cadherin	CGAGCCGCCTGCGCTGCCAC	CGCTGCTCTCCGCTCCCCGC
Vimentin	GACAATGCGTCTCTGGCACGTCTT	TCCTCCGCCTCCTGCAGGTTCTT
β-actin	ATCACCATTGGCAATGAGCG	TTGAAGGTAGTTTCGTGGAT
